# Prevalence and predictors of musculoskeletal health complaints among sedentary, monotonous urban workers: A survey in Bangladesh

**DOI:** 10.1371/journal.pone.0282922

**Published:** 2023-04-21

**Authors:** Mohammad Ali, Md. Abu Bakar Siddiq, Nujaim Khan Pranto, Naheean Hossain Amran, Marium Akter, Marjan Akter Munny, Md. Imran Hossain, Saffat Sabbir Khan, Md. Murad Hossain Mehedi

**Affiliations:** 1 Department of Physiotherapy and Rehabilitation, Uttara Adhunik Medical College and Hospital, Uttara Model Town, Dhaka, Bangladesh; 2 Hasna Hena Pain, Physiotherapy and Public Health Research Center (HPRC), Uttara Model Town, Dhaka, Bangladesh; 3 Department of Physiotherapy, National Institute of Traumatology and Orthopaedic Rehabilitation (NITOR), Sher-E-Bangla Nagar, Dhaka, Bangladesh; 4 Care for Stroke & Geriatric Disabilities, Mohammadpur, Dhaka, Bangladesh; 5 Bangladesh Institute of Health Sciences (BIHS), Darussalam, Dhaka, Bangladesh; 6 Office of the Upazilla Health and Family Planning Officer, Gazipur Sadar, Gazipur, Bangladesh; 7 Mayfair Wellness Clinic, Dhaka, Bangladesh; Prince Sattam Bin Abdulaziz University, College of Applied Medical Sciences, SAUDI ARABIA

## Abstract

**Background:**

Monotonous and sedentary work is significantly associated with the worst health of workers. There is a scarcity of data investigating the musculoskeletal health of sedentary workers working in low-income and middle-income settings. This study aimed to measure the prevalence and predictors of musculoskeletal health complaints (MHC) among Bangladeshi shopkeepers.

**Methods:**

This cross-sectional study was conducted in Dhaka, Bangladesh. Data from 1553 Bangladeshi shopkeepers aged 18 years and above were analyzed. MHC was measured using the musculoskeletal subscale of the subjective health complaints scale. The descriptive analysis helped to compute MHC prevalence and compare the prevalence across groups. Regression analysis revealed the predictors of MHC for the shopkeepers.

**Results:**

The prevalence of MHC among shopkeepers was 58.0%. The prevalence of low back pain was the highest (55.5%), followed by neck pain (48.0%) and upper back pain (43.5%). Regression analysis identified sex (aOR 1.301, CI 0.996 to 1.700), age (aOR 1.405, CI 1.047 to 1.886), body mass index (aOR 0.495, CI 0.397 to 0.617), and substance abuse (aOR 1.998, CI 1.136 to 3.514) as independent predictors of MHC among the shopkeepers. Furthermore, significantly higher odds of MHC have been found among tobacco users (OR 1.234, CI 1.009 to 1.510).

**Conclusion:**

This study revealed a high prevalence of MHC and unhealthy lifestyles among shopkeepers in Bangladesh. Shopkeepers should be provided with better health literacy to follow healthy lifestyles and prevent MHC among this cohort.

## Introduction

Monotonous and sedentary work negatively impacts human health in many ways. However, the world is marching toward more service-related work that forces the working population to engage in tedious, repetitive, and sedentary work. Monotonous work takes a toll on both mental and physical health [1]. Evidence suggests that sedentary workers are more prone to musculoskeletal health complaints (MHC) [2].

MHC includes lower back pain, upper back pain, neck pain, shoulder pain, arm pain, and leg pain are the leading causes of disability that affect 1.7 billion people worldwide [3]. The impact of MHC is heterogeneous and ranges from economic loss to shorter longevity. In 2016, LBP and neck pain management cost $380 billion, about 18% of the GDP in the USA [4]. In addition, evidence suggests that older adults with musculoskeletal health conditions die sooner than those without [5]. MHC is also responsible for office absenteeism, loss of productivity, early retirement, and contributing to years lived with disability [6].

Data suggests that the most significant increases in disabilities caused by MHC have occurred in low-income and middle-income countries (LMICs), including Asia, Africa, and the Middle East [7]. Consequently, MHC is becoming a significant public health problem in LMICs. Much data regarding MHC is required to understand the situation better and discuss the management and prevention strategies [8].

In Bangladesh, previous analyses suggested that the one-month prevalence of MHC among bank employees was 57% [9]. Bangladesh is a densely populated country of LMICs; however, observing a rapidly growing economy as a consequence of more service-related employment in recent decades. Shopkeepers in Bangladesh contribute largely to the economy and work almost every day a week, spending their days in a monotonous sedentary way, thus becoming an excellent sample of a monotonous sedentary worker. This study aimed to examine the prevalence and predictor of MHC among shopkeepers working in an urban Bangladesh setting.

## Materials and methods

### Study design and participants

This cross-sectional study was conducted in Dhaka City Corporation (DCC) area from September 03 to October 06, 2022. Data were collected from male and female full-time Bangladeshi shopkeepers aged 18 years and above currently working in the shops.

### Sample size determination

The actual number of shopkeepers in Bangladesh is mainly unknown. However, we calculate the sample size using a confidence level of 95%, a response distribution of 50%, and a margin of 2.5% error [10]. This formula calculated the minimum sample size to 1537. Previous similar Bangladeshi studies [11–15] found this formula suitable for determining sample size. Approximately 2000 shopkeepers were requested to participate in this study; however, we got consent and data from 1600 participants giving an 80% response rate. After scrutiny, we found 1553 data suitable for analysis. We excluded 47 data due to incomplete and inconsistent answers.

### Ethical issues and informed consent

This study was conducted following the World Medical Association Declaration of Helsinki, ethical principles for medical research involving human subjects [16]. Ethical clearance was taken from the Ethical Review Committee of Uttara Adhunik Medical College (August-2021/2). The Strengthening the Reporting of Observational Studies in Epidemiology guideline for the cross-sectional study was followed strictly throughout the study. Informed consent was taken to the individual interview session after explaining the study’s aim, objective, potential benefit, and dangers. All the participants were also informed about the voluntary nature of the interview and participation.

### The questionnaire

Data were collected using a paper-based questionnaire composed of four parts. The first part of the questionnaire asked a wide range of sociodemographic questions, including sex, age, body mass index (BMI) = weight (kg) / [height (m)]^2^. Overweight was defined as a BMI more than or equal to 25 but less than 30. Obesity was described as a BMI equal to or greater than 30, marital status, education, monthly household income in Bangladeshi Taka (BDT), family type, type of home, and current address. In the second part, participants were asked about their shop-related information, including the type of shop, location, humidification system, working status, experience, and working hours/day. In the third part, participants were asked to provide information about whether they had a chronic disease diagnosis (e.g., hypertension, diabetes, kidney disease, and asthma), whether they were current tobacco users or substance abusers, and whether they regularly performed physical exercise. These questions were answered using dichotomous options (yes/no). The last part of the questionnaire measured musculoskeletal health complaints: shoulder pain, leg pain, arm pain, upper back pain, neck pain, and low back pain. The questions on MHC were based on the musculoskeletal subscale of subjective health complaints produced by Eriksen et al. that estimated MHC experienced in the last 30 days [9, 17, 18]. Participants were asked to rate the occurrence of leg pain, arm pain, upper back pain, neck pain, shoulder pain, and low back pain in 4 categories. The severity of each complaint is rated on a 4-point scale (0  =  none, 1  =  some, 2  =  much, 3  =  severe). Participants who answered "no complaint" or "only once/a little" on all questions were classified as having no musculoskeletal health complaints. Each complaint is scored for the duration (number of days) during the last 30 days. Severity × duration has often been used to obtain a total score (0–90), indicating the degree of the complaint [17]. In this study, participants who complained of at least some pain for 3 days (1 × 3  =  3) in the last month and scored ≥3 were considered as having MHC [18].

### Data collection procedure

Data collectors physically visited shops to collect data. First, nine different locations in DCC were conveniently selected. Second, illegible shopkeepers were requested to participate in this study. Informed consent to collect, analyze and publish their data anonymously was taken before starting an interview. After obtaining permission, one data collector interviewed shopkeepers and fill a paper-based questionnaire. Finally, another data collector rechecked the answered paper. To maintain privacy, participants were interviewed separately in a quiet room in their working area. We only take data of only one shopkeeper from a particular shop to get diversified data. Necessary permission from the in charge or manager of a corresponding shop was taken before starting the data collection.

### Data analysis

We used SPSS version 28.0.0.0 (IBM Corp; USA) software. Chi-square or Fisher’s Exact tests were used to compare categoric variables with and without MHC. To compute the unadjusted odds ratios (ORs) and adjusted odds ratios (aORs) with a 95% confidence interval (CI), multiple logistic regression analyses were performed with MHC as a dependent variable and sociodemographic characteristics, behavior, clinical and service-related factors as predictor variables for MHC. Variables were found statistically significant in the descriptive analysis included in the regression model to compute aORs. The Hosmer-Lemeshow goodness-of-fit test was used to ensure that the models adequately fit the data. P -values ≤0.05 were considered statistically significant.

## Results

### Participants’ general characteristics

Overall, this study analyzed the data of 1553 individuals with the mean ±*SD* age of the participants was 35.90 ±11.4 years. Table 1 depicts the descriptive statistics of the sociodemographic factors and behavioral factors by musculoskeletal health complaints. We had 595 (38.3%) of the respondents who were young adults (18–30 years of age), and 1261 (81.2%) respondents were male. About 59.3% of male participants reported MHC, whereas 52.4% of female respondents reported having MHC. Most participants were overweight (52.2%), Muslim (91.1%), married (70.0%), having an educational level of SSC or lower (58.4%), had a monthly household income ৳ 15000–30000 (48.8%), were from a nuclear family (67.7%), 60.2% were lived in a rented house and lived in the city (62.7%). However, 26.0% and 51.5% reported that they had performed regular physical exercise and were current tobacco users, whereas only 4.6% and 7.5% of participants were substance abusers currently and previously.

**Table 1:** Descriptive analysis: Sociodemographic, behavioral, and clinical factors and MHC.

Variables	Musculoskeletal Health Complaints		Total (%)	p-value
	No (%)	Yes (%)		
**All**	652 (42.0)	901 (58.0)	1553 (100)	
**Sex**				**0.031**
Male	513 (40.7)	748 (59.3)	1261 (81.2)	
Female	139 (47.6)	153 (52.4)	292 (18.8)	
**Age group (years)**				**0.006**
18–30	255 (42.9)	340 (57.1)	595 (38.3)	
31–40	193 (42.0)	266 (58.0)	459 (29.5)	
41–50	109 (34.9)	203 (65.1)	312 (20.1)	
>50	95 (50.8)	92 (49.2)	187 (12.1)	
**Body Mass Index (BMI)**				**<0.001**
Normal	44 (32.1)	93 (67.9)	137 (8.8)	
Overweight	404 (49.8)	407 (50.2)	811 (52.2)	
Obese	204 (33.7)	401 (66.3)	605 (39.0)	
**Religion**				0.869
Islam	594 (42.0)	821 (58.0)	1415 (91.1)	
Hindu	53 (41.4)	75 (58.6)	128 (8.2)	
Others	5 (50.0)	5 (50.0)	10 (0.6)	
**Marital status**				0.497
Married	451 (41.5)	636 (58.5)	1087 (70.0)	
Single	177 (42.3)	241 (57.7)	418 (27.0)	
Divorced/widow	24 (50.0)	24 (50.0)	48 (3.1)	
**Educational Qualification**				0.316
SSC or lower	375 (41.3)	533 (58.7)	908 (58.4)	
HSC	188 (41.2)	268 (58.8)	456 (29.3)	
Graduate and above	89 (47.1)	100 (52.9)	189 (12.1)	
**Monthly household income in thousand (৳)**				0.169
Below 15	164 (41.6)	230 (58.4)	394 (25.3)	
15–30	336 (44.3)	422 (55.7)	758 (48.8)	
31–45	114 (36.9)	195 (63.1)	309 (19.9)	
> 45	38 (41.3)	54 (58.7)	92 (6.0)	
**Family type**				0.200
Nuclear family	430 (40.9)	622 (59.1)	1052 (67.7)	
Joint family	222 (44.3)	279 (55.7)	501 (32.2)	
**Type of home**				0.776
Own	180 (43.5)	234 (56.5)	414 (26.6)	
Rented	383 (41,0)	552 (59.0)	935 (60.2)	
Mess/Hostel	68 (43.0)	90 (57.0)	158 (10.1)	
Others	21 (45.7)	25 (54.3)	46 (3.0)	
**Living location**				0.529
City	415 (42.7)	557 (57.3)	972 (62.7)	
Semi-city	156 (42.3)	213 (57.7)	369 (23.8)	
Village	80 (38.5)	128 (61.5)	208 (13.4)	
**Regular Physical Exercise**				0.728
No	479 (41.7)	669 (58.3)	1148 (74.0)	
Yes	173 (42.7)	232 (57.3)	405 (26.0)	
**Current tobacco user**				**0.041**
No	336 (44.6)	417 (55.4)	753 (48.4)	
Yes	316 (39.5)	484 (60.5)	800 (51.5)	
**Substance abuse**				**0.010**
No	589 (43.1)	777 (56.9)	1366 (88.0)	
Yes	18 (25.4)	53 (74.6)	71 (4.6)	
Previous	45 (38.8)	71 (61.2)	116 (7.5)	
**Shop size**				0.208
Small	278 (44.7)	344 (55.3)	622 (40.0)	
Medium	265 (40.3)	393 (59.7)	658 (42.3)	
Large	109 (39.9)	164 (60.1)	273 (17.5)	
**Type of shop**				0.315
Grocery Shop	77 (38.7)	122 (61.3)	199 (12.8)	
Departmental Store	72 (41.6)	101 (58.4)	173 (11.1)	
Super shop	31 (40.3)	46 (59.7)	77 (5.0)	
Flea and Street market	155 (45.3)	187 (54.7)	342 (22.0)	
Shopping center/ Mall	74 (48.1)	80 (51.9)	154 (10.0)	
Tea Stall	59 (45.4)	71 (54.6)	130 (8.3)	
Jewelers (Gold Maker)	9 (33.3)	18 (66.7)	27 (1.7)	
Others	175 (38.8)	276 (61.2)	451(29.0)	
**Location of shop**				0.426
City Centered	282 (43.5)	367 (56.5)	649 (41.8)	
Suburb	41 (39.8)	62 (60.2)	103 (6.6)	
Roadside	155 (41.8)	216 (58.2)	371 (24.0)	
Remote from City	172 (40.9)	249 (59.1)	421 (27.1)	
Others	1 (12.5)	7 (87.5)	8 (0.5)	
**Humidification system**				0.840
Air Conditioner	130 (42.3)	177 (57.7)	307 (19.7)	
Fan	412 (41.5)	581 (58.5)	993 (64.0)	
Others	110 (43.5)	143 (56.5)	253 (16.2)	
**Working status**				0.517
Owner	438 (43.0)	580 (57.0)	1018 (65.5)	
Salesman	182 (40.0)	273 (60.0)	455 (29.2)	
Others	32 (40.0)	48 (60.0)	80 (5.1)	
**Hypertension**				0.665
No	501 (41.7)	701 (58.3)	1202 (77.3)	
Yes	151 (43.0)	200 (57.0)	351 (22.6)	
**Diabetes**				0.466
No	526 (41.5)	740 (58.5)	1266 (81.5)	
Yes	126 (43.9)	161 (56.1)	287 (18.4)	
**Kidney disease**				0.544
No	619 (42.2)	849 (57.8)	1468 (94.5)	
Yes	33 (38.8)	52 (61.2)	85 (0.5)	
**Asthma**				0.271
No	599 (41.6)	841 (58.4)	1440 (92.7)	
Yes	53 (46.9)	60 (53.1)	113 (7.2)	
Do not know	81 (47.9)	88 (52.1)	169 (10.8)	
**Working experience**				0.803
0–1 years	56 (44.1)	71 (55.9)	127 (8.1)	
2–5 years	223 (40.9)	322 (59.1)	545 (35.0)	
6–14 years	237 (41.5)	334 (58.5)	571 (36.7)	
≥15 years	136 (43.9)	174 (56.1)	310 (20.0)	
**Working hours/day**				0.610
Regular (7–8)	215 (43.8)	276 (56.2)	491 (31.6)	
Over-time (9–12)	364 (41.0)	523 (59.0)	887 (57.1)	
Extra-time (≥13)	73 (41.7)	102 (58.3)	175 (11.2)	

Bold faces are present as a significant variable at a 5% significance level.

Furthermore, 42.3% of the shops were medium in size, 22.0% were flea and street markets, and 29.0% were other types, respectively. Regarding the location of the shop, 27.1% were remote from the city, 64.0% used a fan as a humidification system, 65.5% participants were the owner of the shop, 36.7% participants had 6–14 years of work experience, 57.1% of them were worked over-time (9–12 hours) regularly. However, only 22.6% had hypertension, 18.4% had diabetes, 0.5% had kidney disease, and 7.2% had asthma.

### Prevalence of MHCs

**Fig 1:**
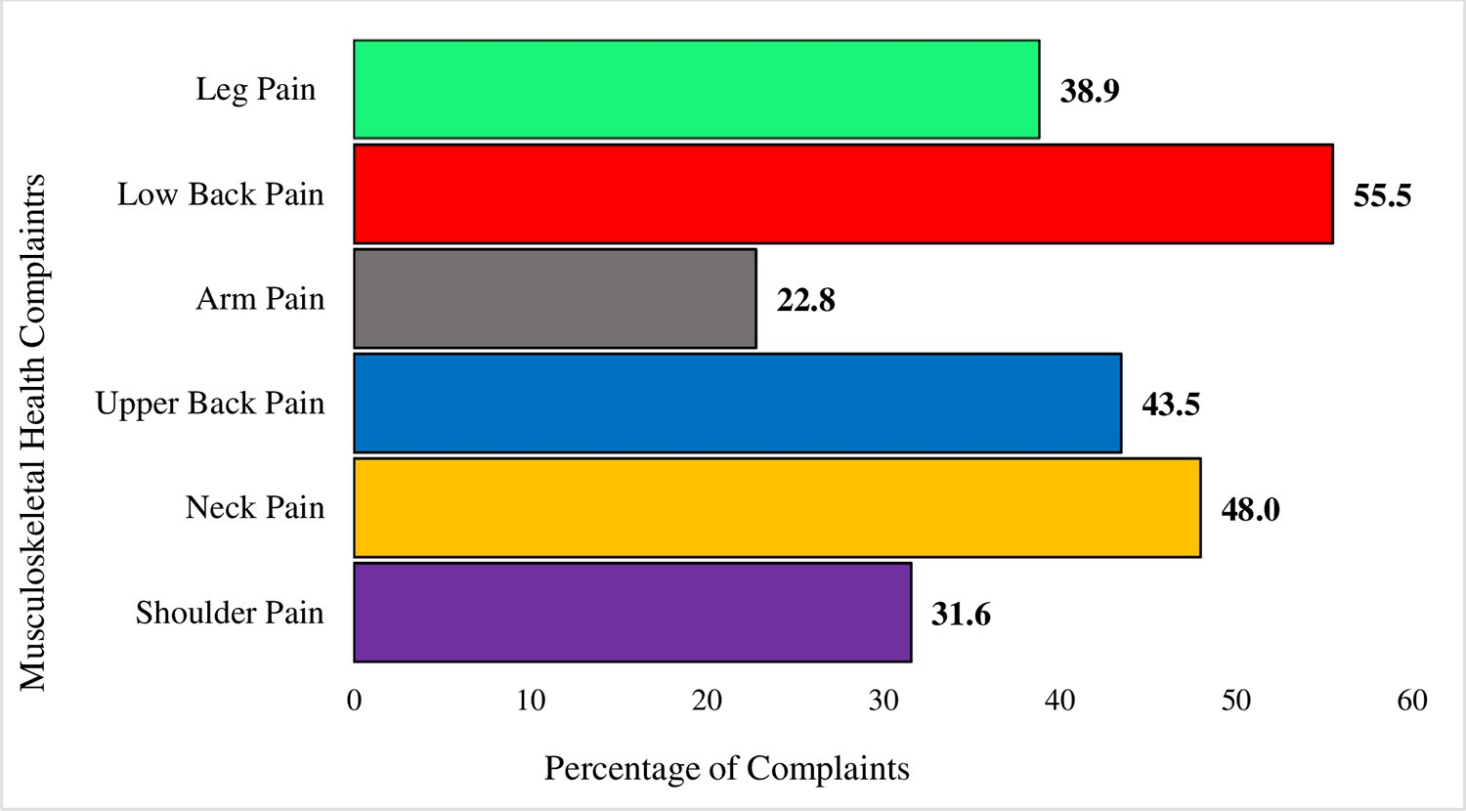
Prevalence of musculoskeletal health complaints among sedentary, monotonous urban workers.

Fig 1 depicts the one-month prevalence of MHCs. Among the six MHCs, the prevalence of low back pain was the highest (55.5%), followed by neck pain (48.0%) and upper back pain (43.5%). Moreover, 38.9% of the employees reported leg pain, whereas 31.6% and 22.8% reported shoulder and arm pain during the last month. A higher prevalence of MHC has been found in males (59.3%, p = 0.031), middle-aged shopkeepers (65.1%, p = 0.006), obese (66.3%, p = <0.001), tobacco users (60.5%, p = 0.041) and substance abusers (74.6%, p = 0.010).

### Result of regression analysis

Higher adjusted odds of MHC have been found in males (aOR 1.301, CI 0.996 to 1.700), age group of 41–50 years (aOR 1.405, CI 1.047 to 1.886), and substance abusers (aOR 1.998, CI 1.136 to 3.514). Furthermore, higher unadjusted odds of MHC have been found in tobacco users (OR 1.234, CI 1.009 to 1.510). However, significantly lower odds of MHC have been found in overweight participants compared to their obese counterparts (aOR 0.495, CI 0.397 to 0.617). Table 2 displays the full results.

**Table 2:** Multiple logistic regression analysis: Predictors of MSD among shopkeepers.

Variables	Unadjusted Odds Ratio	95% Confidence Interval		p-value	Adjusted Odds Ratio	95% Confidence Interval		p-value
**Sex**		Lower	Upper			Lower	Upper	
Female	Reference							
Male	1.325	1.026	1.711	**0.031**	1.301	0.996	1.700	**0.050**
**Age group (years)**								
18–30	Reference							
31–40	1.034	0.808	1.322	0.792	0.977	0.754	1.265	0.858
41–50	1.397	1.052	1.855	**0.021**	1.405	1.047	1.886	**0.023**
>50	0.726	0.522	1.010	0.057	0.654	0.464	0.922	**0.015**
**BMI**								
Normal	1.075	0.723	1.598	0.720	1.052	0.705	1.571	0.804
Overweight	0.513	0.412	0.637	**<0.001**	0.495	0.397	0.617	**<0.001**
Obese	Reference							
**Tobacco use**								
No	Reference							
Yes	1.234	1.009	1.510	**0.041**	1.141	0.916	1.422	0.240
**Substance abuse**								
No	Reference							
Yes	2.232	1.294	3.851	**0.004**	1.998	1.136	3.514	**0.016**
Previous	1.196	0.811	1.764	0.366	1.121	0.744	1.688	0.586

Bold faces are present as a significant variable at a 5% significance level.

## Discussion

This study found that three out of five Bangladeshi shopkeepers complained about MHC. Low back pain was the leading MHC, followed by neck pain, upper back pain, and leg pain. A higher prevalence of MHC was found in males, middle-aged shopkeepers, obese, and tobacco and substance abusers. However, sex, age, BMI, and substance abuse were the predictors of MHC in Bangladeshi shopkeepers.

In addition, this study revealed that more than 90% of the shopkeepers were overweight or obese. More than half of the participants were current tobacco users, and 12% were current or previous substance abusers indicating unhealthy lifestyles among Bangladeshi shopkeepers.

This study found a higher prevalence of MHC among male shopkeepers; however, our previous study found higher odds of MHC among female bank employees [9]. Contrarily, another study conducted in Bangladesh did not find any significant sex-specific differences in MHC prevalence among community dwellers [13]. These study results indicated unmatched findings regarding the sex-specific prevalence of MHC in Bangladesh. Similarly, there is a worldwide scarcity of data that reveals the sex-specific prevalence of MHC among occupational. A large sample size 15-year nationwide population-based cohort study conducted in Taiwan found a similar distribution of MHC among male and female workers in the food and beverage service industry [19]; however, a review indicated that sex differences exist in the presentation of musculoskeletal disease among the adult population [20]. Additional studies among multi-occupational are warranted to reach a conclusion regarding the sex-specific prevalence of MHC in Bangladesh.

Age and BMI are the significant factors that influence MHC. A cohort study that observed 1,759,338 military veterans found a higher prevalence of MHC among obese [21]. Another large sample size cross-sectional survey among the working population revealed similar results [22]. In line with these study findings, the current survey found a significantly higher prevalence of MHC among obese shopkeepers. Furthermore, in line with the results of the present study, our previous studies found that the prevalence of MHC is considerably higher among middle-aged bank employees and community dwellers [9, 13, 23].

A systematic review concluded that smoking hurts the musculoskeletal system [24]. Previous studies also indicated that smoking was associated with lower BMD, increased fracture risk, periodontitis, alveolar bone loss, and dental implant failure [25, 26]. Similar to the previous study in Bangladesh [13], this study found a negative impact of tobacco use on musculoskeletal health.

There is scarce data that investigated the association between substance abuse and MHC. Our survey found a higher prevalence of MHC among current and previous substance abusers. Furthermore, substance abuse was a strong predictor of MHC among the shopkeeper even after adjusting other confounders for MHC. Though further studies are warranted to confirm the result, some literatures indicate that substance abuse is associated with MHC, including LBP [27, 28]. Additional studies are required to validate the results we found in this study.

This study has some limitations that must be acknowledged. First, this cross-sectional study cannot reveal the cause of the association between independent and dependent variables. Second, this study was based on DCC; thus, the result may vary for other urban or rural areas of Bangladesh. Finally, future studies should take data regarding treatment requirements and chronicity of MHC among other occupational.

## Conclusion

This study found a significantly high prevalence of MHC among Bangladeshi shopkeepers. Low back pain was the leading complaint, followed by neck and upper back pain. Almost all the shopkeepers were either overweight or obese, and an increased number of them were tobacco users and substance abusers, indicating that they do not follow a healthy lifestyle. Furthermore, a significantly high percentage of obese, tobacco users and substance abusers were suffering from MHC. Health literacy targeting Bangladeshi shopkeepers might help them when preventing MHC. These findings will also be helpful when discussing treatment strategies for musculoskeletal health complaints among sedentary workers in Bangladesh.

## Supporting information

S1 File Questionnaire. (PDF)

S2 File STROBE checklist. (DOCX)
