# Supplementary material for: Prevalence and predictors of musculoskeletal health complaints among sedentary, monotonous urban workers: A survey in Bangladesh
Source: PLoS One. 2023 Apr 21;18(4):e0282922. doi: 10.1371/journal.pone.0282922 (PMC10120926; doi:10.1371/journal.pone.0282922)
Supplement: S1 File — (PDF) [file pone.0282922.s001.pdf]

Musculoskeletal Health of shopkeepers in Bangladesh

* Required

Socio-demographic factors

1. Birth year *

2. Age *

3. Gender *

Mark only one oval.

- Male
- Female
- Others

4. Height (inch) *

5. Weight (kg) *

6. Religion *

Mark only one oval.

- Islam
- Hindu
- Others

7. Marital status *

Mark only one oval.

- Married
- Single
- Divorced/widow

8. Educational qualification *

Mark only one oval.

- SSC or lower
- HSC
- Graduate and above

9. Household income *

Mark only one oval.

- Below 15 thousand
- 15-30 thousand
- 31-45 thousand
- 45+

10. Family type *

Mark only one oval.

- Nuclear family
- Joint family

11. Current address/location *

Mark only one oval.

- City
- Semi-city
- Village

12. Where do you live? *

Mark only one oval.

- Own house
- Rented house
- Mess/Hostel
- Others

13. Do you perform regular physical exercise? *

Mark only one oval.

- No
- Yes

14. Are you a tobacco user? *

Mark only one oval.

- No
- Yes
- Previous

15. Are you a substance user? *

Mark only one oval.

- No
- Yes
- Previous

Store related information

16. Shop size *

Mark only one oval.

- Small
- Medium
- Large

17. What kind of store is this? *

Mark only one oval.

- Grocery Shop
- Departmental Store
- Super shop
- Flea and Street market
- Shopping center/ Mall
- Tea Stall
- Jewelers (Gold Maker)
- Others

18. Where is your store located? *

Mark only one oval.

- City Centered
- Suburb
- Road-side
- Remote from City
- Others

19. What you use for the humidification system? *

Mark only one oval.

- Air Conditioner
- Fan
- Others

20. What position are you working in this store? *

Mark only one oval.

- Owner
- Salesman
- Others

Comorbidity related factors

21. Have you been diagnosed with hypertension? *

Mark only one oval.

- No
- Yes

22. Have you been diagnosed with diabetes? *

Mark only one oval.

- No
- Yes

23. Have you been diagnosed with kidney disease? *

Mark only one oval.

- No
- Yes

24. Have you been diagnosed with asthma? *

Mark only one oval.

- No
- Yes

Subjective Health Complaints

In last 30 days, how much problem you face for how many days?

25. 1. Shoulder Pain *

Mark only one oval.

- Not At All
- A Little
- Some
- Serious

26. Number of days *

27. 2. Neck Pain *

Mark only one oval.

- Not At All
- A Little
- Some
- Serious

28. Number of days *

29. 3. Upper Back Pain *

Mark only one oval.

- Not At All
- A Little
- Some
- Serious

30. Number of days *

31. 4. Arm Pain *

Mark only one oval.

- Not At All
- A Little
- Some
- Serious

32. Number of days *

33. 5. Low Back Pain *

Mark only one oval.

- Not At All
- A Little
- Some
- Serious

34. Number of days *

35. 6. Leg Pain during physical activity *

Mark only one oval.

- Not At All
- A Little
- Some
- Serious

36. Number of days *

Work-related factors.

37. Working experience *

Mark only one oval.

- 0 -1 years
- 2 - 5 years
- 6 - 14 years
- ≥15 years

38. Working hours/day *

Mark only one oval.

- Regular (7 - 8)
- Over-time (9 - 12)
- Extra-time (≥13)
